# Expression Profiles of Phosphoenolpyruvate Carboxylase and Phosphoenolpyruvate Carboxylase Kinase Genes in *Phalaenopsis*, Implications for Regulating the Performance of Crassulacean Acid Metabolism

**DOI:** 10.3389/fpls.2018.01587

**Published:** 2018-10-30

**Authors:** Chia-Yun Ping, Fure-Chyi Chen, Teen-Chi Cheng, Huey-Ling Lin, Tzong-Shyan Lin, Wen-Ju Yang, Yung-I Lee

**Affiliations:** ^1^Department of Horticulture and Landscape Architecture, National Taiwan University, Taipei, Taiwan; ^2^Department of Plant Industry, National Pingtung University of Science and Technology, Pingtung, Taiwan; ^3^Department of Horticulture, National Chung Hsing University, Taichung, Taiwan; ^4^Department of Biology, National Museum of Natural Science, Taichung, Taiwan; ^5^Department of Life Sciences, National Chung Hsing University, Taichung, Taiwan

**Keywords:** CAM rhythm, orchid, photosynthesis transition, phosphoenolpyruvate carboxylase, phosphoenolpyruvate carboxylase kinase

## Abstract

*Phalaenopsis* is one of the most important potted plants in the ornamental market of the world. Previous reports implied that crassulacean acid metabolism (CAM) orchids at their young seedling stages might perform C_3_ or weak CAM photosynthetic pathways, but the detailed molecular evidence is still lacking. In this study, we used a key species in white *Phalaenopsis* breeding line, *Phalaenopsis aphrodite* subsp. *formosana*, to study the ontogenetical changes of CAM performance in *Phalaenopsis*. Based on the investigations of rhythms of day/night CO_2_ exchange, malate contents and phosphoenolpyruvate carboxylase (PEPC) activities, it is suggested that a progressive shift from C_3_ to CAM occurred as the protocorms differentiated the first leaf. To understand the role of phosphoenolpyruvate carboxylase kinase (PEPC kinase) in relation to its target PEPC in CAM performance in *Phalaenopsis*, the expression profiles of the genes encoding *PEPC* (*PPC*) and *PEPC kinase* (*PPCK*) were measured in different developmental stages. In *Phalaenopsis*, two *PPC* isogenes were constitutively expressed over a 24-h cycle similar to the housekeeping genes in all stages, whereas the significant day/night difference in *PaPPCK* expression corresponds to the day/night fluctuations in PEPC activity and malate level. These results suggest that the *PaPPCK* gene product is most likely involved in regulation of CAM performance in different developmental stages of *Phalaenopsis* seedlings.

## Introduction

*Phalaenopsis*, an epiphytic orchid, is regarded as an obligate crassulacean acid metabolism (CAM) plant because of its succulent leaf with large and highly vacuolated mesophyll cells. Previous studies have indicated that mature plants of *Phalaenopsis* fixed CO_2_ and stored as malate inside the vacuoles at night (Endo and Ikusima, 1989; Ota et al., 1991; Cui et al., 2004; Guo and Lee, 2006). However, it has been reported that CAM orchids at their protocorm or young seedling stages might perform C_3_ or weak CAM pathways (Goh et al., 1984). For instance, no significant day/night changes in the titratable acidity were observed during the protocorm development of *Dendrobium taurinum* (Hew and Khoo, 1980). Chen and Lee (2002) reported that *in vitro* young seedlings of *Phalaenopsis* absorbed more CO_2_ during the light than in the dark. In addition, the *in vitro* young seedlings of *Phalaenopsis* exhibited the carbon isotopic values (δ^13^C) of −21.4‰ to −19.5‰, suggesting a weak CAM photosynthetic pathway (Lo, 2008). These findings imply that an alteration of the photosynthetic pathway from C_3_ to CAM occurred at the early developmental stage of CAM orchids; however, definite proof has not yet been illustrated.

CAM is a water-preserving photosynthetic pathway adaptive in arid environments, in which stomata are closed during the daytime and opened at night In CAM plants, phosphoenolpyruvate carboxylase (PEPC, EC 4.1.1.31) serves as the key enzyme that fixes CO_2_ to phosphoenolpyruvate (PEP) in the cytosol during the nighttime (Osmond and Holtum, 1981; Chollet et al., 1996; Vidal and Chollet, 1997; Nimmo, 2000; Borland et al., 2014), and produces oxaloacetic acid (OAA) stored in the vacuole through the formation of malic or citric acids. In the daytime, decarboxylation of these organic acids increases internal CO_2_ concentration enabling CAM plants to maintain high rates of photosynthesis as the stomata are closed (Winter and Smith, 1996). This feature is an important strategy for the survival of plants in the dry habitat (Silvera et al., 2009; Rodrigues et al., 2013).

Most plants contain several PEPC isoforms encoded by a small gene family that are related to specific physiological functions (Gehrig et al., 1995; Gehrig et al., 2001). In facultative CAM plants, such as the common ice plant (*Mesembryanthemum crystallinum*) and *Kalanchoe* species, the CAM-specific PEPC isoform is induced to fulfill the primary carboxylation and carbon flux while switches to CAM pathway (Cushman et al., 1989; Gehrig et al., 1998). The PEPC isoforms, in addition to a CAM-specific isoform, is important for anapleurotic “housekeeping” or tissue-specific functional roles (Gehrig et al., 1998). Changes in PEPC activity are regulated by phosphorylation of a serine residue of PEPC at night that increases the catalytic activity of PEPC, and reduces the inhibitory effect on PEPC of its allosteric inhibitor, malate (Nimmo G. A. et al., 2001; Taybi et al., 2004). The phosphorylation state of PEPC is catalyzed by PEPC kinase, encoded by *PPCKs* (Chollet et al., 1996; Vidal and Chollet, 1997; Nimmo H. G. et al., 2001). In the common ice plant, the expression of CAM-specific *PPC* and *PPCK* are both induced during the induction of CAM (Cushman et al., 1989; Li and Chollet, 1994; Taybi et al., 2000). In *Clusia minor*, a C_3_-CAM intermediate species, the increased expression of PEPC kinase plays an interesting role in regulating CAM performance (Borland and Griffiths, 1997). Altogether, examining the relationship between the expression of CAM-specific *PPC* and *PPCK* could be a great strategy to illustrate the developmental changes in CAM orchids.

In orchids, protocorms are tuber-shaped structures derived from germinating embryos (Arditti, 1992). The protocorm is a transitional structure, which subsequently produces the shoot and roots, resulting in the formation of a young seedling. If the protocorms or young seedlings of *Phalaenopsis* perform C_3_ type metabolism, the developmental stage from which the plants onset the shift from C_3_ to CAM is of interested. To elucidate the progression from C_3_ to CAM photosynthetic pathway in *Phalaenopsis*, we investigated the expression of *PPC* and *PPCK* genes, the profiles of day/night CO_2_ exchange, malate contents and the PEPC activity of different developmental stages. During the past decade, commercial production of *Phalaenopsis* as potted flowering plants has increased massively in the world (Griesbach, 2000; U.S. Department of Agriculture [USDA], 2017). A better understanding the regulation in CAM performance will provide insights into the micropropagation and cultivation of *Phalaenopsis*.

## Materials and Methods

### Plant Materials

**FIGURE 1:**
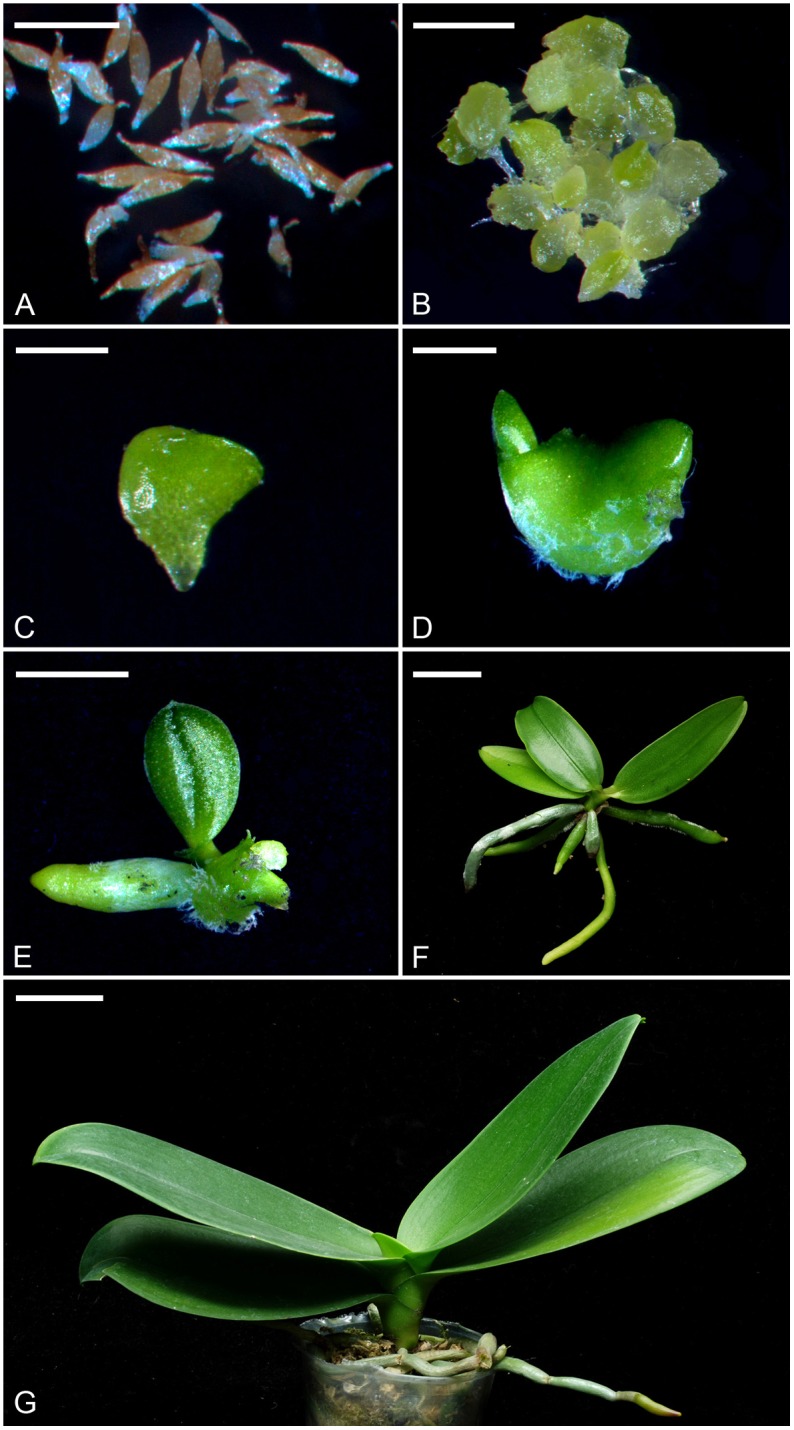
The seeds, protocorms and seedlings of *P. aphrodite* subsp. *formosana* at different developmental stages (stage 0 to stage 6). **(A)** Stage 0: mature seeds. Bar = 0.25 mm. **(B)** Stage 1: the embryo swelled and turned green after 2 weeks of culture. Bar = 1 mm. **(C)** Stage 2: the protocorm of diameter 1–3 mm could be observed after 4 weeks of culture. Bar = 2 mm. **(D)** Stage 3: the protocorm with a shoot apical meristem could be observed after 6 weeks of culture. Bar = 4 mm. **(E)** Stage 4: the protocorm had differentiated the first true leaf and root after 10 weeks of culture. Bar = 1 cm. **(F)** Stage 5: the seedling at the time for taking out of flasks after 32 weeks of culture. Bar = 2 cm. **(G)** Stage 6: a 3-year-old mature seedling represented the mature stage that was planted in a 9 cm pot with sphagnum moss. Bar = 4 cm.

The mature plants of *Phalaenopsis aphrodite* subsp. *formosana* were maintained in a greenhouse with the pad-and-fan cooling system at National Museum of Natural Science, Taichung, Taiwan. To ensure good pod set and seed quantity, flowers at anthesis were hand-pollinated immediately. Mature seeds were harvested at 150 days after pollination for *in vitro* cultures. For *in vitro* seed germination, we used the 1/4-strength macro-elements (i.e., KNO_3_, NH_4_NO_3_, KH_2_PO_4_, MgSO_4_⋅7H_2_O and CaCl_2_⋅2H_2_O) and full-strength micro-elements (i.e., FeSO_4_⋅7H_2_O, Na_2_⋅EDTA, MnSO_4_⋅4H_2_O, ZnSO_4_⋅7H_2_O, CuSO_4_⋅5H_2_O, KI, CoCl_2_⋅6H_2_O, H_3_BO_3_, NaMoO_4_⋅2H_2_O) of Murashige and Skoog (MS) medium (Murashige and Skoog, 1962), supplemented with 10 g⋅L^−1^ sucrose (Sigma–Aldrich Co., St. Louis, MO, United States), 1 g⋅L^−1^ peptone (Merck KGaA, Darmstadt, Germany) and 2.2 g⋅L^−1^ Gelrite (Sigma–Aldrich Co.). The pH value of the media was adjusted to 5.7 with 1 N KOH prior to autoclaving at 121°C and 1.2 kg⋅cm^−2^ for 15 min. When the first true leaf and root emerged, the developing protocorms were subcultured onto the culture medium containing 1/2-strength macro-elements and full-strength micro-elements of MS medium, supplemented with 10 g⋅L^−1^ sucrose, 1 g⋅L^−1^ peptone and 2.2 g⋅L^−1^ Gelrite. The cultures were placed at the growth chamber at 30 μmol⋅m^−2^⋅s^−1^ (daylight fluorescent tube FL-20D/18, 20W, China Electric Co., Taipei) and 26 ± 2°C with a 12-h daylength (0600 to 1800 h). Different developmental stages were shown and described in Figure 1.

### Measurement of Diurnal CO_2_ Exchange

Since developing protocorms (the stages 1–3) were too small to measure the photosynthetic rate using LI-COR’s portable photosynthesis systems, we measured the changes of CO_2_ concentrations within the culture tubes for representing their photosynthetic profiles. For obtaining the diurnal CO_2_ change curves *in vitro*, one gram of protocorms were inoculated onto the culture medium in a culture tube (120 mm tall × 13 mm with 11 mm inside diameter) sealed with the aluminum foil. The cultures were placed in the growth room at 30 μmol⋅m^−2^⋅s^−1^ (daylight fluorescent tube FL-20D/18, 20W, China Electric Co., Taipei) and 26 ± 2°C with a 12-h daylength (0600 to 1800 h). The air within the sealed culture tube was collected by a 1-mL syringe every 2 h during a 24-h period, and the CO_2_ concentration was determined by IR-analyzer (UNOR 610, Maihak AG, Hamburg, Germany). Each data point was measured by the accumulation of CO_2_ concentration for 2 h within the culture tube. Before the first measurement, the aluminum foil was removed from the culture tube to balance the CO_2_ concentration from ambient air for 5 min, then the culture tube was sealed with a rubber stopper for 2 h. After each measurement, the rubber stopper was opened to balance the CO_2_ concentration from ambient air. Three replications were performed in each stage, and each replicate contained the measurement of three tubes. The empty tubes without protocorms were used for CO_2_ measurements (340 ppm) for calibration.

After the leaf differentiation (stages 4, 5, and 6), gas exchange parameters were measured on the middle part of the top second leaf of a plant by a portable Infra Red Gas Analyzer (LI-COR 6400, LI-COR, Lincoln, NE, United States) with a 2 cm^2^ leaf chamber every 2 h during a 24-h period. Photosynthetic responses were measured at leaf temperature of 30 ± 2.5°C with 350 μmol⋅mol^−1^ CO_2_ supplied at a flow rate of 200 μmol⋅s^−1^. In the stages of 4 and 5, young seedlings grown in flasks (six seedlings per flask) were take out and the roots were wrapped in the moistened sphagnum moss for measurements. The leaf in the stage 4 was too small to fit a 2 cm^2^ leaf chamber, and the lead area was estimated by LI-3100C Area Meter for correction of leaf area measurement according to the LI-6400 manual. In the stage 6, 3-year old seedlings grown in the greenhouse were measured. In these experiments, the cultures and seedlings were placed at the growth chamber at 30 μmol⋅m^−2^⋅s^−1^ (daylight fluorescent tube FL-20D/18, 20W, China Electric Co., Taipei) with a 12-h daylength (0600 to 1800 h). The sampled leaf was placed in the leaf chamber for 20 min before the data readings were recorded. The environmental conditions in the leaf chambers were controlled as close to those in the growth chamber as possible to reduce the variations between the sampled leaves. In this study, the completely randomized design was used, and the measurements were performed with three replications, and each replication represented the average of six seedlings of each stage.

### Measurement of Malate Levels

About 0.2 g tissue from developing protocorms or leaves at each stage was sampled every 2 h during a 24-h period. In stage 5 and 6, the middle part of the top second leaf of a plant was collected for measurement. The samples were ground in a mortar with 5 mL of distilled water. The crude extracts were heated at 90°C water bath for 30 min, then cooled at room temperature. The malate content of the cooled extract was measured according to the method of Möllering and Malt (1974). The concentration of malate was determined using standard curves. For the analysis of titratable acidity, 4 mL of the supernatant was titrated with 0.01 N NaOH solution to the end point of pH value at 8.3. The amount of NaOH was used to calculate the concentration of the titratable acid, expressed as micromoles H^+^ per gram fresh weight. In this study, the completely randomized design was used, and the measurements were performed with three replications, and each replication represented the average of six seedlings of each stage. The significantly different by *t*-test between day and night in the same stage were indicated by asterisk (*P* < 0.05).

### Assay of PEPC Activity

PEPC was extracted from developing protocorms and leaves of 0.1 g of each stage were sampled every 2 h during a 24-h period. In stage 5 and 6, the middle part of the top second leaf of a plant was collected for measurement. The sample was homogenized with 1.5 ml extraction buffer (50 mM Tris-HCl, pH 7.0, 10 mM MgCl_2_, 1 mM EDTA, 5 mM DTT, 2.5% PVPP and 10% glycerol) according to the method of Ku et al. (1999). The homogenate was centrifuged at 17709 × *g* for 15 min at 4°C. PEPC activity of the supernatant was measured according to Krömer et al. (1996). Protein concentration was determined by using Bio-Rad protein assay reagent (Bradford, 1976). In this study, the completely randomized design was used, and the measurements were performed with three replications, and each replication represented the average of six seedlings of each stage. Significantly differences between day and night in the same stage, as identified by a *t*-test, were indicated by asterisks (*P* < 0.05).

#### Cloning of cDNAs of *PaPPCK* of *P. aphrodite* subsp. *formosana*

Total RNA was extracted from the leaves of seedlings at stage 5 using RNeasy Plant Mini Kit (Qiagen, Hilden, Germany) and modified as Gehrig et al. (2000). For the double-stranded cDNA synthesis, 1 μg RNA was reverse-transcribed using the SuperScript^TM^ III kit (Invitrogen, Carlsbad, CA, United States) according to the manufacturer’s instruction. The synthesized cDNA fragments and the *PaPPCK* degenerate primers designed from the conserved amino acid sequences in the expressed sequence tag (EST) library of *Phalaenopsis* (FC Chen, unpublished) were used in PCR experiments. The amplified fragment containing the partial sequence of *PaPPCK* that showed high sequence similarity to PPCK was identified by using a blastx algorithm in a BLAST search against the NCBI database (National Center for Biotechnology Information, GenBank). The internal gene-specific primers were designed from the partial sequence of *PaPPCK* for 5′ and 3′ rapid amplified cDNA ends (RACE) using SMART^TM^ RACE cDNA amplification kit (Clontech, Palo Alto, CA, United States). The full-length cDNA for *PaPPCK* was obtained by PCR amplification using the PaPPCK-F and PaPPCK-R primers. All the primers used were listed in Supplementary Table S1.

### Real-Time PCR Analysis

Real-time PCR was performed using ABI PRISM 7300 Sequence Detection System (Applied Biosystems, Waltham, MA, United States) with SYBR Green PCR Master Mix (Applied Biosystems) for time-course transcript measurements. Seeds (stage 0), protocorms (stages 1–3) or the top second leaves of seedlings at different stages (stages 4–6) were collected every 2 h for total RNA isolation and cDNA synthesis. The condition of real-time PCR was 95°C for 10 min, then 40 cycles of amplification (95°C for 15 s, 60°C for 1 min, 72°C for 30 s). The real-time PCR experiments were repeated at least three times. The gene-specific primers for real-time PCR for two *PPC* genes and one *PaPPCK* gene were designed using the Primer Express 2.0 (Applied Biosystems). The *Actin* gene of *P. aphrodite* subsp. *formosana* was used for normalization. All the primers used were listed in Supplementary Table S1.

### Reverse-Transcription PCR Assay

For total RNA isolation and cDNA synthesis, the top second leaf of mature plants of *P. aphrodite* subsp. *formosana* were collected every 2 h at the growth chamber at 30 μmol⋅m^−2^⋅s^−1^ (daylight fluorescent tube FL-20D/18) with a 12-h daylength (0600 to 1800 h). Primers specific for two PEPC isogenes, i.e., *PPC1* (PEQU07008) and *PPC2* (PEQU14315) in RT-PCR amplification were designed using Primer3 Input (v. 0.4.0)^1^. The condition for amplification was one cycle of 94°C for 5 min; 35 cycles of 95°C for 30 s, 55°C for 30 s, and 72°C for 30 s; and a final elongation at 72°C for 5 min. The *Actin* gene of *P. aphrodite* subsp. *formosana* was used as the positive control.

### Phylogenetic Analysis

The PaPPCK sequence was analyzed by using a BLASTX algorithm in a BLAST search against the NCBI database to find the closest sequence matches in the database. Multiple alignments of amino acid sequences was performed using the ClustalW program (Thompson et al., 1994) in BioEdit Ver. 7.0.0 (Hall, 1999). Phylogenetic relationships were estimated using MEGA Ver. 4 with Neighbor-joining (NJ) analysis. The scale bar indicates a genetic distance for 0.1 amino acid substitutions per site. Every branch was supported by bootstrap analysis for 1000 replications (values of more than 70% are shown below the branches). Calcium-dependent protein kinase (CDPKs) sequences were used as the outgroup. Accession numbers of genes used for alignment and phylogenetic analyses in this study were listed in Supplementary Table S2.

## Results

### Photosynthetic Characteristics at Different Developmental Stages

**FIGURE 2:**
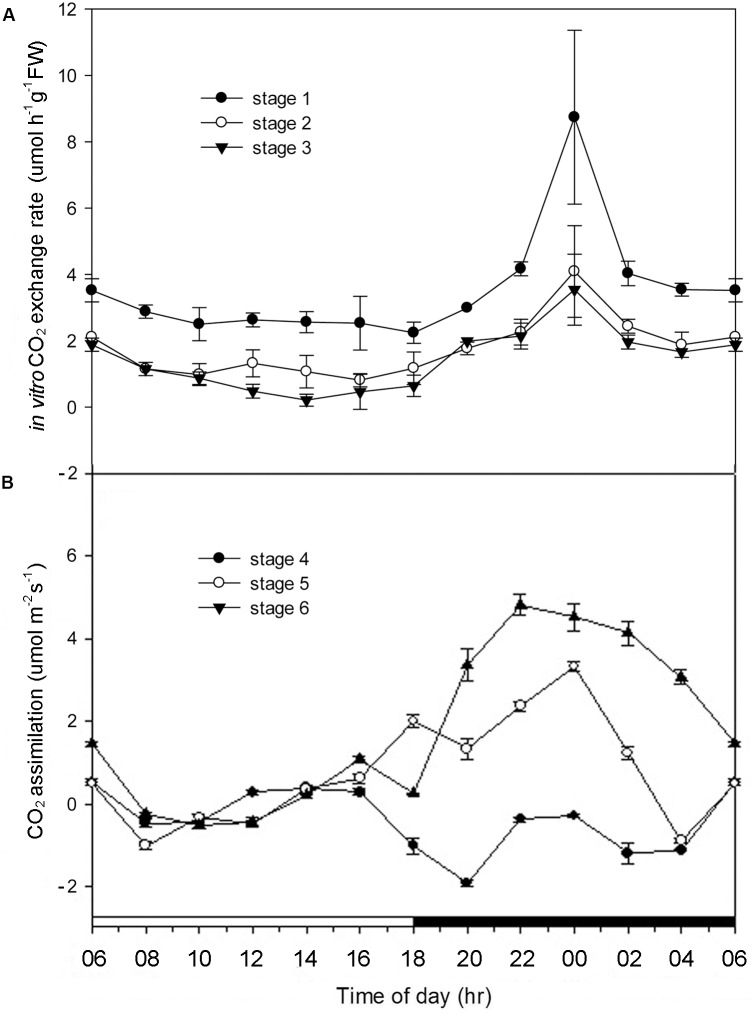
Alterations of CO_2_ exchange rate in *Phalaenopsis* at different developmental stages. **(A)** CO_2_ exchange rate of *Phalaenopsis* in culture tubes measured by IR-analyzer from stages 1 to 3 during a 24-h period. **(B)** CO_2_ assimilation of *Phalaenopsis* at stages 4–6 measured by LI-COR’s portable photosynthesis system during a 24-h period. Error bars represent standard error (SE) for three independent replications with the 3 mean of three culture tubes. The black bar on the *x*-axis represent the dark period.

In developing protocorms, higher CO_2_ concentrations within culture tubes were detected during the dark period than the light period, indicating the lack of CAM expression (Figure 2A). By stage 4, only a small amount of net CO_2_ assimilation were detected during the light period, but no net CO_2_ assimilation was detected during the dark period (Figure 2B). The typical net CO_2_ assimilation rhythm of CAM was first observed by stage 5, which showed a major net CO_2_ assimilation during the dark period.

### Malate Contents and PEPC Activities at Different Developmental Stages

**FIGURE 3:**
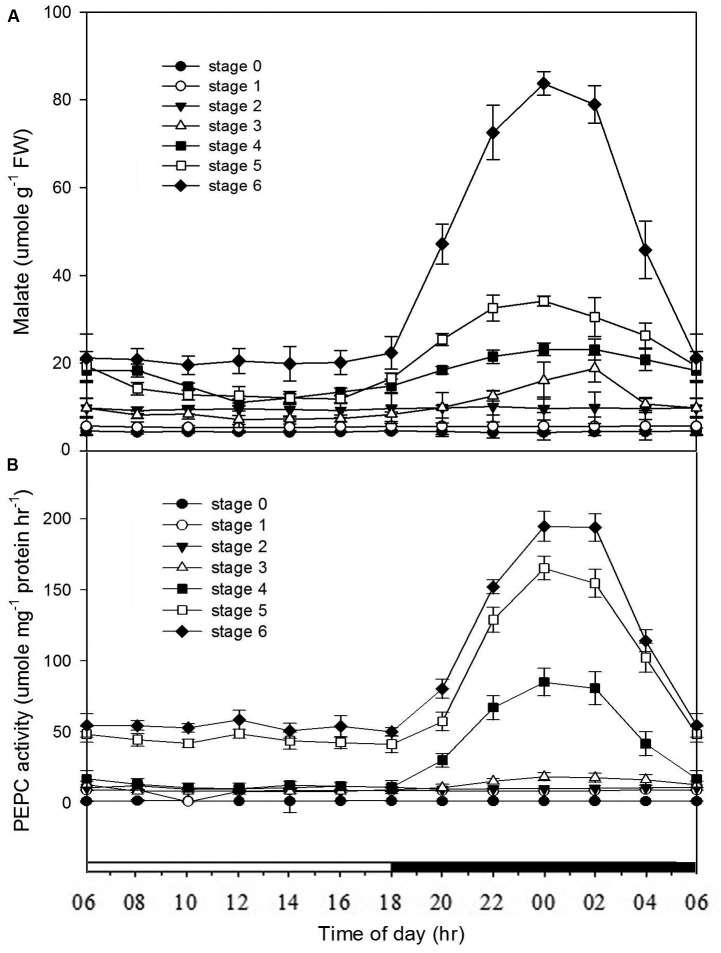
Changes of malate contents and PEPC activities in *Phalaenopsis* at different developmental stages. Day/night changes in **(A)** malate content and **(B)** PEPC activity in different developmental stages (stages 0–6). Malate and PEPC activity were detected in the mature seed (stage 0), the enlarged embryo (stage 1), the protocorm (stages 2 and 3) and the different developmental ages of leaf (stages 4–6). Error bars represent standard error (SE) for three independent replications with the mean of six samples.

From stages 0 to 2, no diurnal differences in the malate contents were observed. By stage 3, the noteworthy diurnal fluctuations in the malate contents first appeared, and the diurnal fluctuations were becoming noticeable as the seedlings becoming mature (stages 4–6) (Figure 3A). In developing protocorms and seedlings, the diurnal fluctuations in PEPC activity were similar to those in malate contents (Figure 3B).

### Characterization of PEPC Isoforms in *Phalaenopsis*

The whole genome sequence dataset of *Phalaenopsis equestris* revealed two isoforms of *PPC* (i.e., PEQU07008 and PEQU14315) occurred in its genome (Cai et al., 2015), while only one copy of *PPC* (AJ300742) was found in the EST library of *P. aphrodite* subsp. *formosana* (previously known as *P. amabilis*) (Tsai et al., 2013). In this study, molecular phylogenetic analysis indicated that PEQU07008 and AJ300742 were nested in *PPC1* clade (the CAM-related), while PEQU14315 was nested in *PPC2* clade (the anapleurotic) (Supplementary Figure S1).

### Isolation and Characterization of *PaPPCK* cDNA

**FIGURE 4:**
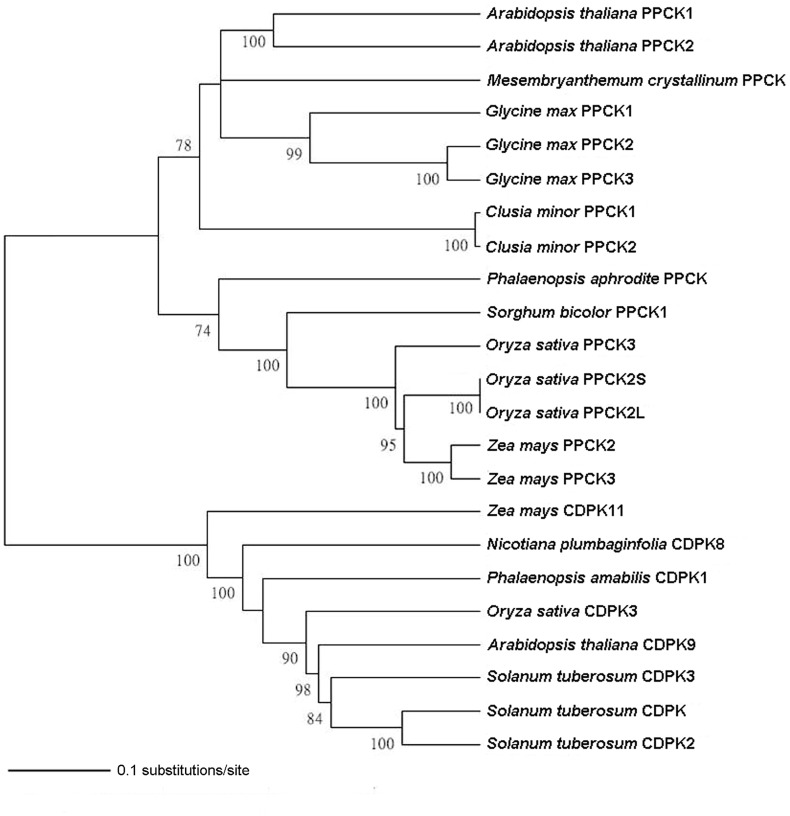
Phylogenetic analysis of the PaPPCK protein. The phylogenetic relationship is conducted using Neighbor-Joining with 1000 bootstrap replicates (bootstrap values < 70% are not shown). The scale bar indicates a genetic distance for 0.1 amino acid substitutions per site. Calcium-dependent protein kinase (CDPKs) sequences are used as the outgroup. The accession numbers of genes are given in Supplementary Table S2.

Full-length of *PaPPCK* cDNA was successfully cloned from *P. aphrodite* subsp. *formosana* using a combination of RT-PCR and RACE strategies. The GenBank accession numbers of *PaPPCK* sequence was 2100748. The cDNA contains an open reading frame of 843 bp, flanked by 5′- and 3′-untranslated sequences of 35 and 233 bp, respectively. The cDNA of *PaPPCK* encodes a protein of 281 amino acids that demonstrates 61∼64%, 61%, and 57% sequence identity to three rice PPCKs, ice plant PPCK, and *Kalanchoe fedtschenkoi* PPCK, respectively (Supplementary Figure S2). The molecular phylogenetic analysis among PaPPCK and several other PPCK orthologs revealed that PaPPCK clustered with the same group of monocots (Figure 4).

### Expression and Abundance of *PPC* Isogenes and *PaPPCK*

**FIGURE 5:**
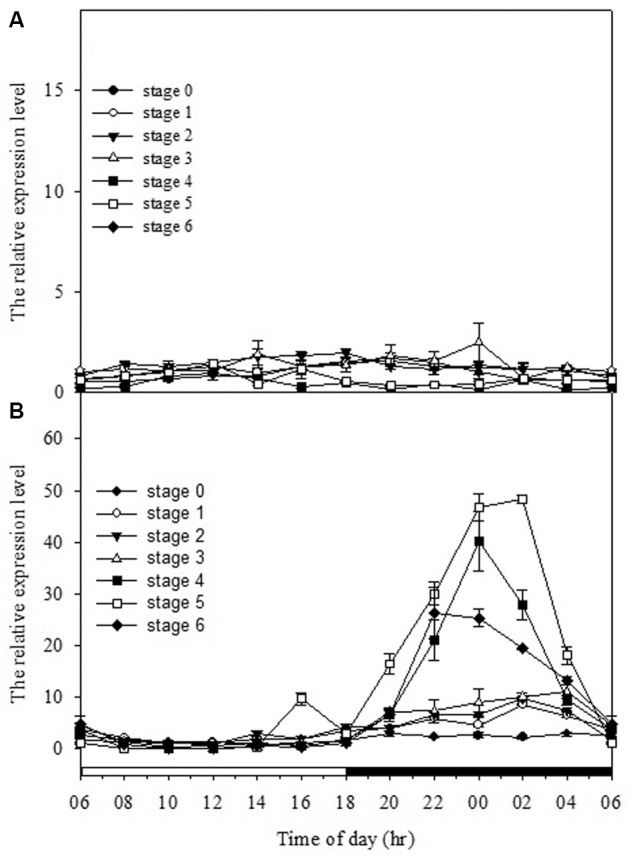
Day/night changes in levels of **(A)** *PPC1* (AJ300742) and **(B)** *PaPPCK* (2100748) in different developmental stages (stages 0–6). Relative transcript levels are measured by real-time PCR with *Actin* gene of *P. aphrodite* subsp. *formosana* as loading control. Error bars represent standard error (SE) for three independent replications. The black bar on the *x*-axis represents the dark period.

To gain the further insight into the relationships among the CO_2_ assimilation, malate content, PEPC activity and gene expression patterns at different developing stages, the mRNA levels of *PPC* and *PaPPCK* were analyzed every 2 h over a 24 h period by quantitative real-time PCR analysis (Figure 5). As expressions of *PPC* isogenes in different developmental stages were measured, no obvious day/night fluctuations was observed, and their expressions were constitutive and rather constant. From stages 4 to 6, the expression pattern of *PaPPCK* showed a typical diurnal/nocturnal rhythm.

## Discussion

In *Phalaenopsis*, the nocturnal CO_2_ uptake was not observed until stage 5 (Figure 2). During stages 1–3, no obvious depletion of CO_2_ *in vitro* was detected, and the increase of CO_2_ concentration within the culture tubes from light to dark periods may reflect the elevated respiration rate of high-energy requirements in developing protocorms (Figure 2A). Differences between day/night malate and PEPC activity was first observed at stage 3 and the breadth of day/night fluctuation enlarged as the seedlings developed (Figure 3). These results implied a progressive shift from C_3_ to CAM as the protocorms differentiated the first leaf in *Phalaenopsis* (from stages 3–5). The age-dependent expression of CAM has been reported in a number of plants. In *M. crystallinum*, CAM performance is modulated ontogenetically by a genetic developmental program, although high salinity could rapidly induce CAM expression in young plants (Cushman et al., 1990; Cushman and Bohnert, 1996). Several CAM species, such as *Ananas comosus, Kalanchoe fedtschenkoi*, *Peperomia*, and *Clusia* species, have demonstrated that a progression from C_3_ to CAM occurred as the leaves becoming matured (Jones, 1975; Holthe et al., 1987; Zotz and Winter, 1996; Vaasen et al., 2006; Winter et al., 2008). During the transitional phase from C_3_ to CAM, the photosynthetic features (e.g., no significant nocturnal CO_2_ uptake but having malate accumulation in the stage 4 seedlings) were similar to CAM-cycling in which plants kept their stomata closed and accumulation organic acids by recapturing the respiratory CO_2_ during the night (Holthe et al., 1987; Ting et al., 1996). CAM-cycling strategy allows the maintenance of photosynthetic capacity as well as water saving during drought or in the dry season (Harris and Martin, 1991). In *Phalaenopsis*, upon germination, the embryo first develops into a protocorm before forming a plantlet. Since the differentiation of first root takes about 8 weeks after germination to uptake water efficiently, CAM-cycling may help protocorms to tolerate water deficit.

Previous reports have shown that the performance of CAM could be transcriptionally regulated at the mRNA amounts of PEPC as plants became mature or in response to stress conditions (Cushman et al., 1990; Haasg-Kerwer et al., 1992; Borland et al., 1996). To determine if the ontogenetic difference in CAM performance in *Phalaenopsis* was reflected by differences in the expression of *PPC* transcripts, we measured the expression and abundance of two *PPC* isogenes in different developmental stages. In orchids, molecular phylogenetic analysis of *PPC* genes indicated that *PPCs* can be separated into two clades that reflected their different functions, i.e., the CAM-related *PPC1* and the anapleurotic function *PPC2*, respectively (Silvera et al., 2014; Deng et al., 2016). Our previous study (Ping et al., 2010) as well as the results in this study demonstrated that both *PPC1* (the CAM-related) and *PPC2* (the anapleurotic) expressed in all developmental stage regardless of the time point (Figure 5 and Supplementary Figure S3), suggesting that the day/night rhythm of PEPC activity is not likely controlled at the abundance of both *PPC* transcripts but another factor.

In addition to the transcriptional control of PEPC activity, the posttranslational control by PEPC kinase also plays an important role in CAM performance (Hartwell et al., 1999; Taybi et al., 2004). The activation of PEPCs in CAM is regulated through a reversible phosphorylation by PEPC kinase (Vidal and Chollet, 1997; Nimmo, 2000, 2003). PEPC kinase is encoded by a small gene family involved in a variety of functions for the precise regulation of PEPC activity (Sullivan et al., 2004; Fukayama et al., 2006; Shenton et al., 2006). In *Phalaenopsis*, so far only one gene encoding PEPC kinase has been found. In general, *PaPPCK* increases its expression levels as the seedlings grew up. High levels of *PaPPCK* transcripts were detected during the dark period and extremely low amounts during the light period, particularly in the plants of stage 3 (Figure 5B). In stage 5, the increased magnitudes of malate accumulation and PEPC activity were not as high as the level of *PaPPCK* expression (Figures 3, 5). The existence of a protein inhibitor of PEPC kinase may provide an additional layer of control over malate accumulation by PEPC activity (Nimmo G. A. et al., 2001). Indeed, the apparent day/night difference in *PaPPCK* expression corresponds to day/night fluctuations in PEPC activity and malate levels. In *C. minor*, a C_3_-CAM intermediate species, CAM performance is also achieved through the shift in *PPCK* expression (Borland and Griffiths, 1997). In facultative CAM species, e.g., *K. fedtschenkoi* and *M. crystallinum*, the phosphorylation of PEPC is primarily controlled by the abundance of the *PPCK* transcript, and hence facilitates the nocturnal CO_2_ uptake (Hartwell et al., 1999; Taybi et al., 2000). It is known that PPCK contains a protein kinase domain without regulatory regions indicated that its activity appears to be controlled primarily at the level of expression (Nimmo H. G. et al., 2001; Nimmo, 2003). In *Phalaenopsis*, the distinctive expression pattern of *PaPPCK* leads us propose that PaPPCK likely plays a critical role in regulating the phosphorylation state of PEPC during the night to CAM performance.

## Conclusion

Our results demonstrated a progressive shift from C_3_ to CAM as the protocorms differentiated the first leaf in *Phalaenopsis* seedlings by measuring the profiles of day/night CO_2_ exchange, malate contents, PEPC activity and the expression of *PPC* isogene*s* and *PaPPCK*. Notably, in *Phalaenopsis* seedlings, the day/night fluctuations in PEPC activity and the expression of *PaPPCK* as well as CO_2_ uptake was coincident well along the developing stages. Since *PPC* isogenes were constitutively expressed over a 24-h cycle, *PaPPCK* may be involved in regulating CAM performance in *Phalaenopsis*.

## Author Contributions

Y-IL conceived the study. Y-IL, F-CC, T-SL, and W-JY designed the study. C-YP, F-CC, and T-CC performed molecular analyses. C-YP performed qPCR and biochemical experiments. C-YP and H-LL performed photosynthesis analyses. C-YP, Y-IL, W-JY, and F-CC wrote the paper. All the authors read and approved the final manuscript.

## Conflict of Interest Statement

The authors declare that the research was conducted in the absence of any commercial or financial relationships that could be construed as a potential conflict of interest.

## Footnotes

1 http://primer3.ut.ee

## Supplementary Material

FIGURE S1 Phylogenetic analysis of PEPC amino acid sequences using Neighbor-joining method described above in the phylogenetic analysis. The other sequences of PEPC obtained from GenBank were added to the analysis by referring to Deng et al. (2016). The *PEPC* genes from *Phalaenopsis* are denoted by arrows. Branch numbers indicated bootstrap support (1000 replicates). Bootstrap values < 70% were not shown.

FIGURE S2 Alignment of deduced PaPPCK amino acid sequence with PPCKs. The accession numbers of genes were given in Supplementary Table S2. Black and shadow represented identities and similarities respectively. At, *Arabidopsis thaliana*; Ft, *Flaveria trinervia*; Kf, *Kalanchoë fedtschenkoi*; Mc, common ice plant; Os, rice; Pa, *Phalaenopsis aphrodite*; Sb, *Sorghum bicolor*; Zm, maize.

FIGURE S3 Day/night changes in levels of transcripts for two *PEPC* genes, i.e., *PPC1* (PEQU07008 and AJ300742), *PPC2* (PEQU14315), and *Actin* as control for equal RNA input and RT-PCR conditions in mature leaves of *P. aphrodite* subsp. *formosana*.

Table S1 Primer pairs used in this study.

Table S2 PPCK and CDPK sequences used for conducting the amino acid alignment and phylogenetic analysis.
