# Supplementary figures and images for: Expression Profiles of Phosphoenolpyruvate Carboxylase and Phosphoenolpyruvate Carboxylase Kinase Genes in Phalaenopsis, Implications for Regulating the Performance of Crassulacean Acid Metabolism
Source: Front Plant Sci. 2018 Oct 30;9:1587. doi: 10.3389/fpls.2018.01587 (PMC6218735; doi:10.3389/fpls.2018.01587)

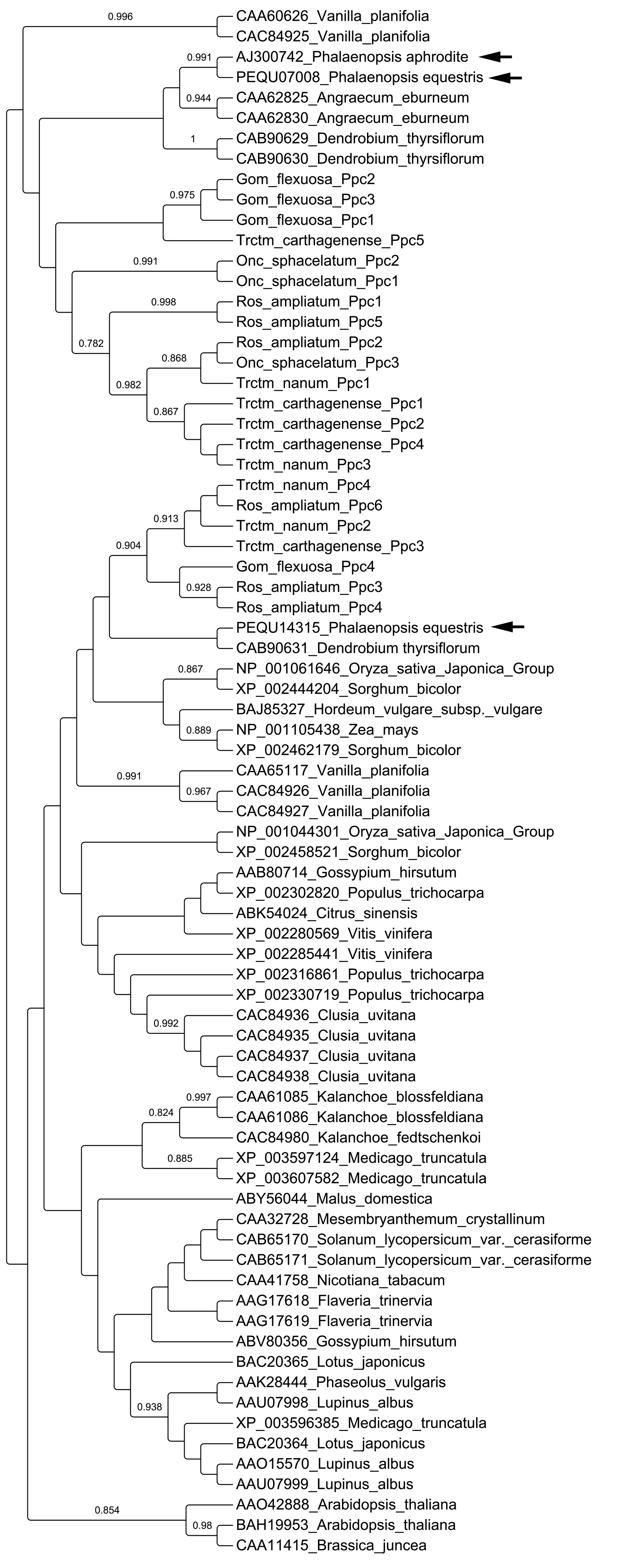

Supplement: FIGURE S1 — Phylogenetic analysis of PEPC amino acid sequences using Neighbor-joining method described above in the phylogenetic analysis. The other sequences of PEPC obtained from GenBank were added to the analysis by referring to Deng et al. (2016). The PEPC genes from Phalaenopsis are denoted by arrows. Branch numbers indicated bootstrap support (1000 replicates). Bootstrap values < 70% were not shown. [file Image_1.JPEG]

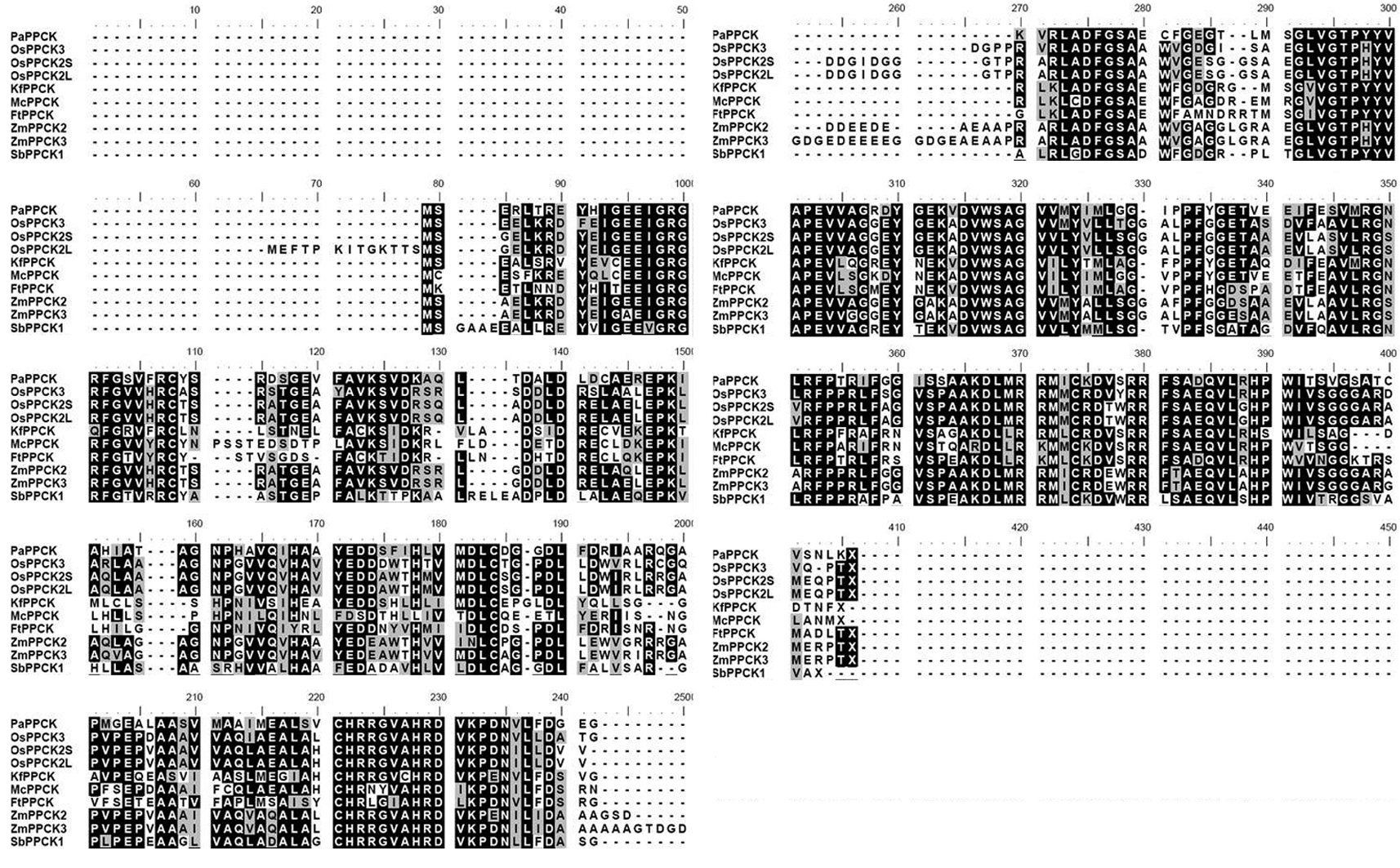

Supplement: FIGURE S2 — Alignment of deduced PaPPCK amino acid sequence with PPCKs. The accession numbers of genes were given in Supplementary Table S2. Black and shadow represented identities and similarities respectively. At, Arabidopsis thaliana; Ft, Flaveria trinervia; Kf, Kalanchoë fedtschenkoi; Mc, common ice plant; Os, rice; Pa, Phalaenopsis aphrodite; Sb, Sorghum bicolor; Zm, maize. [file Image_2.JPEG]

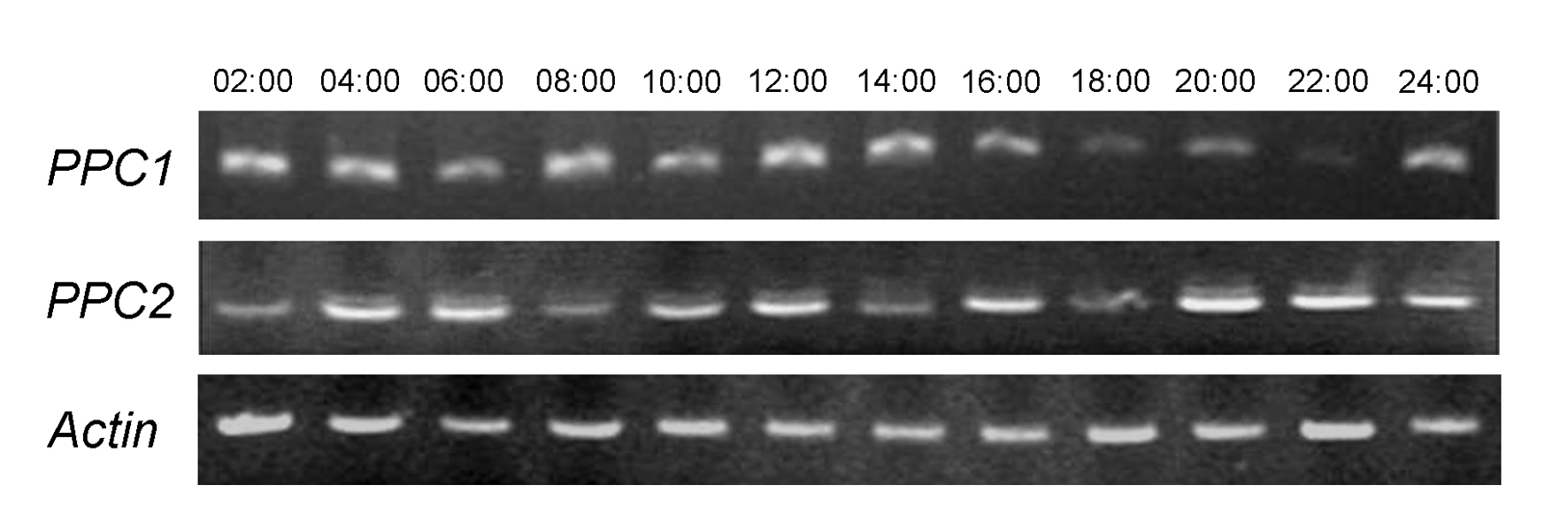

Supplement: FIGURE S3 — Day/night changes in levels of transcripts for two PEPC genes, i.e., PPC1 (PEQU07008 and AJ300742), PPC2 (PEQU14315), and Actin as control for equal RNA input and RT-PCR conditions in mature leaves of P. aphrodite subsp. formosana. [file Image_3.JPEG]
