# Supplementary material for: Expression Profiles of Phosphoenolpyruvate Carboxylase and Phosphoenolpyruvate Carboxylase Kinase Genes in Phalaenopsis, Implications for Regulating the Performance of Crassulacean Acid Metabolism
Source: Front Plant Sci. 2018 Oct 30;9:1587. doi: 10.3389/fpls.2018.01587 (PMC6218735; doi:10.3389/fpls.2018.01587)
Supplement: Table S1 — Primer pairs used in this study. [file Table_1.DOC]

Supplementary Table S1. Primer pairs used in this study.

| Purpose and primer name | Primer sequence (5’ to 3’) |
| --- | --- |
| Cloning  *PaPPCK*-F3  *PaPPCK*-R1  Reverse transcription PCR | GGCGGCGATCATGGAGGCGTTGTCTGTT  CTCCCCATAAAACGGAGG |
| *PPC*-F1 | CAGAAGCATGGCAGGATAAGAAAT |
| *PPC*-R1 | CAGCGCCACATAGATAACTCAAAC |
| *PaPPCK*-F1 | ACGCCCTTGACCTTGATT |
| *PaPPCK*-R1 | CTCCCCATAAAACGGAGG |
| *18S rDNA*-F1 | TGGCCTACTATGGTGGTG |
| *18S rDNA*-R1 | GGCATCGTTTATGGTTGA |
| Real-time PCR |  |
| *PPC*-F2 | GGAAGCCGACCATCTAAACG |
| *PPC*-R2 | CCGGAACCAAGCCAAACA |
| *PaPPCK*-F2 | GACGCCCTTGACCTTGATTG |
| *PaPPCK*-R2 | CGGCGGTGGCGATGT |
| *PhalActin*-F1 | GCTGTCTTCCCCAGCATTGT |
| *PhalActin*-R1 | CAACCATTACTCCGGTATGACG |
