# Supplementary material for: Expression Profiles of Phosphoenolpyruvate Carboxylase and Phosphoenolpyruvate Carboxylase Kinase Genes in Phalaenopsis, Implications for Regulating the Performance of Crassulacean Acid Metabolism
Source: Front Plant Sci. 2018 Oct 30;9:1587. doi: 10.3389/fpls.2018.01587 (PMC6218735; doi:10.3389/fpls.2018.01587)
Supplement: Table S2 — PPCK and CDPK sequences used for conducting the amino acid alignment and phylogenetic analysis. [file Table_2.DOC]

Supplementary Table S2. *PPCK* and *CDPK* gene sequences used for conducting the amino acid alignment and phylogenetic analysis.

| Plant species | Gene name | GenBank Accession No. |
| --- | --- | --- |
| *Arabidopsis thaliana* | *PPCK1* | AF162660 |
|  | *PPCK2* | AF358915 |
|  | *CDPK9* | NM_112932 |
| *Clusia minor* | *PPCK1* | AY478419 |
|  | *PPCK2* | AY478420 |
| *Flaveria trinervia* | *PPCK* | AB065100 |
| *Glycine max* | *PPCK1* | AY144180 |
|  | *PPCK2* | AY144182 |
|  | *PPCK3* | AY373033 |
| *Mesembryanthemum crystallinum* | *PPCK1* | AF158091 |
| *Nicotiana plumbaginifolia* | *CDPK8* | AJ699160 |
| *Oryza sativa* | *PPCK2L* | AB233453 |
|  | *PPCK2S* | AB233452 |
|  | *PPCK3* | AB234234 |
|  | *CDPK3* | AY271296 |
| *Phalaenopsis amabilis* | *CDPK1* | EF555574 |
| *Phalaenopsis aphrodite* | *PPCK* | This study |
| *Solanum tuberosum* | *CDPK* | AF115406 |
|  | *CDPK2* | AF418563 |
|  | *CDPK3* | AF518003 |
| *Sorghum bicolor* | *PPCK1* | DQ386731 |
| *Zea mays* | *PPCK2* | AY911414 |
|  | *PPCK3* | AY911415 |
|  | *CDPK2* | EU968057 |
|  | *CDPK11* | AY301062 |
